# Age Difference in Roles of Perceived Social Support and Psychological Capital on Mental Health During COVID-19

**DOI:** 10.3389/fpsyg.2022.801241

**Published:** 2022-02-23

**Authors:** Shiyue Cao, Yue Zhu, Pei Li, Wei Zhang, Cody Ding, Dong Yang

**Affiliations:** ^1^Faculty of Psychology, Southwest University, Chongqing, China; ^2^Department of Educational Psychology, University of Missouri–St. Louis, St. Louis, MO, United States

**Keywords:** perceived social support, psychological capital, mental health, adolescence, COVID-19

## Abstract

Due to the outbreak of the Coronavirus Disease-2019 (COVID-19) pandemic and consequent confinement measures, young people are vulnerable to mental health problems. The current study compared a group of 440 young adolescents (10–12 years) and a group of 330 emerging adults (18–25 years) to investigate the extent to which perceived social support and psychological capital (PsyCap) were differentially associated with mental health problems. Participants were asked to report their current psychosocial adaptation status during the COVID-19 pandemic, and data were collected via online questionnaires during a relatively severe period of COVID-19 in China. Results of the multi-group path analysis indicated that the effect of perceived social support on mental health problems was mediated by PsyCap for young adolescents, but not for emerging adults. These results were discussed with respect to the mechanism of how social support and PsyCap serve as protective mental health factors for youth in the context of the COVID-19 pandemic.

## Introduction

The impact of Coronavirus Disease 2019 (COVID-19) has been expanding from social economy to individual health since its outbreak at the end of 2019. Apart from the fatal threat of the virus itself, an increasing body of research has focused on mild-to-severe mental health problems caused by the strike of COVID-19. Anxiety, depression, and stress are common mental health problems experienced during the COVID-19 pandemic. A high incidence of mental health disorders among adults has been reported during this pandemic (Fan et al., 2021). Additionally, young adolescents without mature cognitive and life skills seem more vulnerable to the mental health effects of the COVID-19 pandemic (Guessoum et al., 2020).

Social support has been recognized as a salient factor during the COVID-19 pandemic. Social support, defined as either psychological or material resources, is regarded as a protective resource that helps individuals cope with stress (Cohen, 2004). The “buffering-effect model” suggests protective effects of social support in stressful situations. For instance, the conservation of resources (CORs) theory states that social support serves as an external supplement of resources that people can rely on to avoid psychological distress in stressful circumstances (Hobfoll et al., 2016). Perceived social support is reported to be associated with reduced symptoms from a series of mental disorders, such as major depressive disorder (MDD), posttraumatic stress disorder (PTSD), and generalized anxiety disorder (GAD), after stressful events (Price et al., 2018). During the COVID-19 pandemic, social support from family could have potentially alleviated depression symptoms due to the social lockdown condition (Mariani et al., 2020). Moreover, it has been shown that a high level of perceived social support from multiple sources is associated with better physical and psychological health (Fang et al., 2020). Thus, social support may be of great importance for protecting one from mental health problems, especially in a crisis, such as the prolonged COVID-19 pandemic.

In addition to social support, internal strengths are another potential protective factor. Psychological capital (PsyCap) is defined as a collection of malleable psychological strengths that support individuals to live adaptively, even when enduring stress or adversity (Luthans and Youssef-Morgan, 2017). Multiple constructs have been proposed as the components of PsyCap, such as hope, resilience, confidence (self-efficacy), positive attribution (optimism), positive emotions, and self-esteem (Luthans et al., 2015). Accumulating research on PsyCap derived from the fields of organizational behavior (OB), human resource management (HRM), and human resource development (HDR) and has more recently entered the fields of relationship, education, sport, and physical and psychological health (Luthans and Youssef-Morgan, 2017). Since the theoretical mechanism underlying the concept of PsyCap was largely drawn from stress research, it is suitable for explaining resilience in stressful situations, such as the COVID-19 pandemic, in the current study (Turner et al., 2014).

A positive correlation exists between these two external and internal protective factors. More specifically, the effect of social support on mental health may be mediated by PsyCap. When individuals perceive more social support, their positive PsyCap level will be higher (Yarcheski and Mahon, 2016). PsyCap plays a positive role in promoting mental health. Higher PsyCap may enable individuals to have highly adaptive and capable coping mechanisms so that they can successfully overcome depression and anxiety and reduce psychological symptoms (Duan, 2016). The research of Han shows that individuals' social support and PsyCap are significantly correlated with their mental health. PsyCap plays an intermediary role in the prediction of social support on mental health (Han et al., 2017).

Interestingly, however, the effects of perceived social support and PsyCap may vary among children of different age groups due to their developmental characteristics. Adolescents are in a critical developmental stage, both physically and psychologically. Hormonal changes, internal reactivity, and social factors act together to shape adolescents' mental state and wellbeing. Changes in hormones increase sensitivity to negative emotions and increase the probability of experiencing depression (Buchanan et al., 1992). Socially, interaction with peers or other people aside from their families becomes a more important part of their lives (Smetana et al., 2015). This maturation process, unfortunately, was abruptly interrupted for many young adolescents due to the COVID-19 pandemic. Social distancing policies and school shutdowns resulted in more than 220 million children and adolescents being confined to their homes in China (Wang et al., 2020). These confinement measures have significantly reduced peer interactions and outdoor activities for adolescents. Children and young adolescents have been found to be more suspectable to psychological distress, partially because of decreased opportunities to socialize as they did pre-pandemic (Saurabh and Ranjan, 2020).

From another perspective, young adolescents may have not yet developed sufficient competency in relying on PsyCap to deal with adversity without external support. Although regarded as a psychological resource that benefits mental health, PsyCap gradually builds up through adolescence (Wang et al., 2016). As they get older, adolescents may reduce their reliance on perceived social support and utilize their internal strength (Archer et al., 2019). Since PsyCap in young adolescents has not yet fully developed as an internal strength to rely on during adversity (King et al., 2020), they are highly dependent on social support, which predicts more positive mood and fewer emotional problems. Emerging adults (typically refers to ages 18–25), however, have developed a certain degree of internal strength to rely on during adversity, although social support is still an important protective factor. This internal strength may be a result of adult identity, or “taking responsibility for themselves” (Arnett, 2015), and more mature cognitive abilities and life skills to cope with various adversities (Larsen and Luna, 2018). For example, previous research of adolescents who had experienced the Ya'an earthquake has indicated that, for young adolescents, more obtained social support is associated with lower levels of reported PTSD symptoms after a disaster (Zhou et al., 2018). In contrast, for emerging adults, a meta-analysis on the association between social support and psychosocial outcomes found significant heterogeneity of effects caused by age: samples of younger adolescents showed larger effects (Heerde and Hemphill, 2018). Similarly, frequency of interaction with family or friends was not found to be associated with depression in emerging adults (Werner-Seidler et al., 2017), implying diminished effects of social support in comparison with younger adolescents.

These age differences regarding reliance on social support and PsyCap to foster mental health among adolescents and emerging adults may be more salient during the COVID-19 pandemic due to unprecedented social distancing policies and school shutdowns. However, this has not yet been fully examined. Therefore, it is imperative to explore protective factors for the youth's mental health and clarify age differences in mechanisms to serve as a guideline for implementing preventative strategies, particularly, for those with limited access to mental health care during the pandemic.

## Hypotheses

In order to examine the age differences that concern associations between PsyCap, social support, and mental health status during the pandemic, we hypothesized a mediation model moderated by age using data collected during a relatively serious period of the COVID-19 pandemic (6–9 March, 2020). First, we hypothesized that for both young adolescents (10–12 years old) and emerging adults (18–25 years old), perceived social support and PsyCap were expected to be positively associated with mental health status, and PsyCap may play a mediating role. Second, we hypothesized a larger effect size of PsyCap among emerging adults than young adolescents. Although PsyCap may lead to better mental health status, its enhancing effect on mental health was expected to be minor among young adolescents during the COVID-19 pandemic. In other words, we expected that as adolescents grow older, they would utilize more internal strength in addition to social support to enhance their mental health status because emerging adults have more agency and the ability to confront stressful situations on their own.

## Method

### Participants

The sample of the current study consisted of participants in two age groups who were recruited separately. We excluded the patients who took less than 2 min to complete the questionnaire as their responses might not be valid. The final sample for the younger group (10–12 years old) comprised of 440 young adolescents (195 men and 245 women); the sample for the older group (18–25 years old) comprised of 330 emerging adults (138 men and 192 women). In addition, we also collected demographic information about their residential location and living arrangements. Among the younger participants, 83.4% participants were from rural areas; 42.7% participants were the only child in the family; 67.1% participants lived with both their parents, and 10.9% participants lived with other living arrangements. As for emerging adults, 96% participants were undergraduate or graduate students, and 97.6% participants were single.

### Procedure

Data were collected online via web link. Responses to questionnaire items were anonymous, and participation was voluntary. Participants were informed that this study aimed at investigating the mental health status among the public during the COVID-19 pandemic. All the results of this study would be used for academic research and help to improve the public policies. Participation and responses to the questionnaire were confidential.

To collect data for young adolescents, we invited the head teachers of various classes to help deliver the questionnaires to parents via an online survey website (www.wjx.cn). The teachers instructed the parents to monitor their kids to complete the questionnaire. Informed consent and assent forms were completed before the study. The study was approved by the university research review committee.

To collect data from emerging adults, participants were recruited via a research network. Individuals who were willing to participate in the study were sent a link to complete the questionnaires via an online survey website (www.wjx.cn). The informed consent form was obtained before participants completed the study. The study was approved by the university research review committee.

### Measures

To assess the participants' reaction to the COVID-19 pandemic, we specifically asked the participants to respond to the questionnaire concerning the pandemic. In other words, we asked the participants to think about the effects of the pandemic on their perceived social support, PsyCap, and their mental health status.

#### Psychological Capital

For the younger group, PsyCap was assessed using the PsyCap Scale of School Children (Peng et al., 2014) based on the Chinese version of Luthans' positive psychological scale. The response scale ranged from 1 to 6 (very dissatisfied to very satisfied), with higher scores indicating higher levels of PsyCap. The Cronbach's alpha of this scale in our study was 0.92. For the older group, we used Positive Psychological Capital Questionnaire (PPQ) developed by Kuo (2010), since we slightly tailored it to be used in a more general context (e.g., original item “At present time, I am energetically pursuing my work goal”; modified item “At present time, I am energetically pursuing my study or work goal”). The reliability and validity tests in college students indicate a good quality of this scale, and the Cronbach's alpha of items was 0.90 in the current study.

#### Social Support

For the younger group, perceived social support was assessed by five items of a subjective social support dimension from a revised version of the Social Support Rating Scale (SSRS) (Xiong and Ye, 2013), which was particularly designed for young adolescents. The participants rated each item on a 4-point scale, with one indicating little perceived social support and four indicating much perceived social support. The Cronbach's alpha was 0.66 in the current sample.

For the older group, perceived social support was assessed by four items taken from SSRS. This scale, developed by Shuiyuan (1994), has been widely used in clinical assessment and academic research, and good reliability and validity were reported. The scale used in the younger group was a version for young adolescents based on this scale. The Cronbach's alpha of the items used in the current study was 0.71.

#### Mental Health Problems

Mental health problems were assessed using 21 items of the short form of the Depression Anxiety Stress Scales (DASS-21) concerning one's state in the last week (Henry and Crawford, 2005). It has three subscales: depression, anxiety, and stress. The participants rated each item on a 4-point scale; 0 indicated that the item did not apply to the participant and 3 indicated that the item completely applied to the participant. This scale has been used in many studies with Western and Chinese participants. It has been suggested that the general score of DASS-21 should be used rather than its three subscales (Zanon et al., 2021), especially when the study is focused on general mental health status among individuals. Accordingly, the total score of the instrument was used as the indicator of mental health. The Cronbach's alpha was 0.97 for this scale.

Considering all the variables that were assessed in one questionnaire battery, we checked the common method variance (CMV) using the unmeasured latent method construct (ULMC) approach (Richardson, 2009). The fits of the models with and without method construct were not significantly different (ΔTFI and ΔCFI <0.1; ΔRMSEA <0.05), suggesting no serious issues of CMV in the study.

#### Demographic Variables

In addition to the study variables, we collected the following demographic variables for young adolescents: gender, residential location (rural areas or urban areas), single-child status (yes or no), and living arrangement (“live with both parents,” “live with mother or father,” or “live without parents”). For emerging adults, demographic variables included gender and educational level (“below junior high school,” “senior high school,” “undergraduate degree,” or “postgraduate degree or above”).

### Statistical Analysis

Because different measures were used in the two age groups, we standardized the sum score of variables, combined them into one data file, and then encoded the age of the younger group as 1 and the older group as 2. Second, a descriptive analysis was performed on the key variables. Due to the fact that variables were in a moderately non-normal distribution (Finney and DiStefano, 2013), the bootstrap method estimation was used to analyze non-normal data in subsequent analyses (Brown, 2015). Then, we conducted pathway analyses to test the effects of perceived social support and PsyCap on mental health, setting age as the group variable to compare effects. We controlled for gender in the analysis. The analyses were performed using Mplus 8.0 in SPSS 24.

## Results

### Descriptive Analysis

The descriptive analysis was conducted based on standardized data, and thus we mainly interpreted the results of skewness. As shown in Table 1, the results indicated that all participants generally displayed a relatively high level of perceived social support (skewness = −0.33), and PsyCap (skewness = −0.61). Negatively skewed distributions of DASS-21 scores implied a higher level of mental health among all participants. For example, the skewness of perceived social support was negative (−0.81) for the younger group, indicating that more adolescents perceived relatively high levels of social support. By contrast, the skewness of perceived social support was positive (0.31) for the older group, indicating that a slightly larger portion of participants in this group perceived relatively low levels of social support.^1^

**Table 1:** The skewness of perceived social support, psychological capital, and mental health problems in two age groups.

	**Younger group (*n* = 440)**		**Older group (*n* = 330)**	
**Variables**	**Skewness**	**SE**	**Skewness**	**SE**
PSS	0.811	0.116	0.313	0.134
PC	−0.964	0.116	−0.133	0.134
MHP	2.326	0.116	1.670	0.134

*PSS, perceived social support; PC, psychological capital; MHP, mental health problems;*

*SE, standardized error of skewness*.

Table 2 shows the correlations among these three variables. As expected, perceived social support and psychological capital were positively correlated, and these two variables were both negatively correlated with mental health problems.

**Table 2:** Correlations among perceived social support, psychological capital, and mental health problems across groups.

	**Younger group**			**Older group**		
**Variables**	**1**	**2**	**3**	**1**	**2**	**3**
1. PSS	1	0.419^**^	−0.157^**^	1	0.349^**^	−0.389^**^
2. PC		1	−0.109^**^		1	−0.396^**^
3. MHP			1			1

^*^p <0 .05;

** *p < 0.01;*

^***^p < 0.001. PSS, perceived social support; PC, psychological capital; MHP, mental health problems.

### Path Analysis

**Figure 1:**
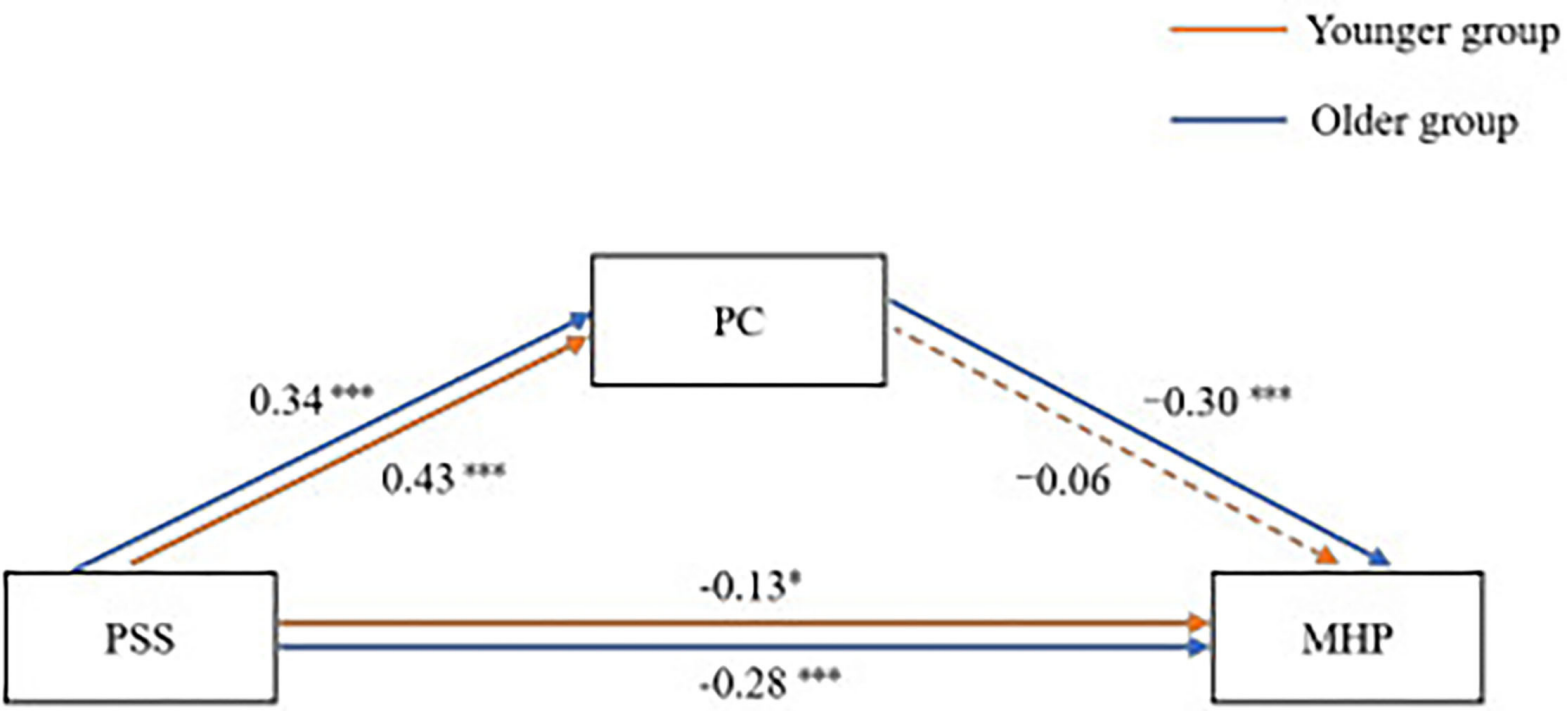
Path analysis by two age groups. *p < 0.05; **p < 0.01; ***p < 0.001. PSS, perceived social support; PC, psychological capital; MHP, mental health problems. The dashed pathway was not significant.

We conducted path analysis by age group (younger vs. older group). Given that there were only three variables involved, the path model was saturated, and model fit was perfect by necessity (CFI/TFI = 1.00 and RMSEA/SRMR = 0.00). Notice that the key point of the path model in this study was to compare how the effect size of path coefficients differed by age group rather than examining the model fit. Results of the pathway analysis are shown in Figure 1. Perceived social support was positively associated with PsyCap, and these two variables were both negatively associated with mental health problems. However, the effect of PsyCap on mental health problems was not significant in the younger group. This was further supported by the comparison of the effect difference, which showed that the effect of PsyCap in the older group was significantly larger than that in the younger group (effect difference = 0.24, *p* = 0.011). The coefficients of pathways in the two models are shown in Table 3. The results seem to suggest that both perceived social support and PsyCap impact mental health for emerging adults, while PsyCap does not significantly affect mental health for young adolescents.

**Table 3:** Path coefficients by age groups.

	**Young adolescents (effect 1)**		**Young adults (effect 2)**	
	**Coefficient**	**SE**	**Coefficient**	**SE**
PSS → PC	0.425^***^	0.043	0.337^***^	0.051
PSS → MHP	−0.131^*^	0.052	−0.286^***^	0.052
PC → MHP	−0.055	0.052	−0.295^***^	0.052

* *p < 0.05;*

^**^p < 0.01;

*** *p < 0.001. PSS, perceived social support; PC, psychological capital; MHP, mental health problems*.

## Discussion

The main purpose of the current study was to examine age differences regarding the benefits of perceived social support and PsyCap on the mental health of adolescents and emerging adults during the COVID-19 pandemic. Specifically, we tested differential effects of perceived social support and PsyCap as protective factors for mental health to buffer the negative effects of the pandemic among two age groups. As somewhat expected, perceived social support helps to buffer against mental distress for all participants, while PsyCap only exerts a significant effect among the older group.

Protective effects of perceived social support found in the current study were aligned with previous research (Cohen, 2004). The benefits of social support have been repeatedly observed in adolescents. Social support can help to fulfill psychological needs (e.g., “relatedness” in self-determination theory) and buffer against the risk of psychological disorders induced by stressful events related to post-disaster maladaptive symptoms (Turner et al., 2014). The results of this study also show that the perceived social support of young adolescents and emerging adults is conducive to PsyCap.

It is important to note that the link between social support and mental health might be more prominent in young adolescents. Previous studies examined the association between social support and psychosocial outcomes and have implied larger effect sizes of social support for younger adolescents (Heerde and Hemphill, 2018). A recent study conducted during COVID-19 also found a buffering effect of social support on anxiety and depression symptoms in Chinese adolescents (14–18 years old) (Qi et al., 2020). Social and family support perceived by young adolescents (9–17 years old) was associated with more positive lifestyles, which would improve mental health (Zhu et al., 2021). Since younger adolescents are characterized by high dependence on sources of social support, less access to social interaction due to social distancing in the COVID-19 pandemic predisposes them to maladaptive symptoms (Guessoum et al., 2020). During the epidemic period, a sufficient amount of social support is particularly important for their psychological health (Vélez et al., 2016). The social support provided could provide adolescents with psychological resources to actively deal with the epidemic and alleviate the negative effects of stress on their bodies and mind. Although the influence of parents' social support on mental health is weakened with age, it still serves as a significant protective factor.

Seemingly surprising but consistent with our hypothesis, the effect of PsyCap on mental health between young adolescents and emerging adults was different. That is, PsyCap has little effect on mental health in young adolescents, while a significant effect on young adults. PsyCap utilizes positively selective attention and interpretation and mitigates the prevalence of negativity bias (Youssef-Morgan and Luthans, 2015). Such positivity, according to the broaden-and-build theory, broadens a person's attention and thus makes them more likely to notice, attach, and accept available resources, which then improves mental health (Fredrickson and Branigan, 2005). Emerging adults, who have rapidly developing self-identities, self-evaluations, brains, and bodies, are more likely to deal with problems independently. They can resort to internal strength when encountering stresses (Skinner and Zimmer-Gembeck, 2016). As we found, PsyCap shows a significant effect on maintaining mental health as adolescents grow older, although social support still serves as a protective factor under stressful conditions. Consequently, PsyCap and social support can be regarded as two components of “resource caravans” for emerging adults, as they work together to achieve better protection of individuals' mental health (Hobfoll et al., 2016).

In addition, a minor result worth mentioning is that the younger group seemed to have a higher level of perceived social support than the older group. One possible explanation could be the difference in the subjective perception process. Research has shown that participants report stronger levels of objectively received social support when they subjectively perceive that support is needed (Melrose et al., 2015). As discussed before, young adolescents have more need for social support than adults. Therefore, even if they receive as much social support as the older group, the younger group could perceive it as more.

## Implications

The study has two implications for adolescents' mental health during the pandemic. First, social support is undoubtedly important for maintaining strong mental health during COVID-19. As the WHO has advocated, more social support would help individuals confront stress during the pandemic (WHO, 2020). For both young adolescents and emerging adults, additional social support is quite necessary when facing increased social isolation and confinement during the COVID-19 pandemic (Lee et al., 2020; Loades et al., 2020). Teachers, social workers, and policy-makers should take extra steps to make sure adequate social support (both material and emotional) is provided. However, particular attention should be paid to younger adolescents in ensuring that they have sufficient social support so they can be more effective in confronting problems or challenges. Second, it is important to help adolescents to cultivate PsyCap to combat against negative effects of social isolation and confinement due to the pandemic. Although emerging adults may have naturally developed a relatively higher level of PsyCap than the younger adolescents, it is still not necessarily sufficient in such a pandemic. More educational programs for adolescents should target cultivating PsyCap and coping skills to boost positive mental health before a potential crisis.

## Limitations

Some limitations of the current study need to be noted. First, the present study is based on cross-sectional data, due to the particular context of COVID-19. Although the participants were explicitly asked about their situation under this pandemic, longitudinal studies are imperative to examine the effect of change during the pandemic. Second, due to the lockdown measures, we could not monitor the data collection process. Although we investigated CMV and no seriously methodological problems were observed, there might still be inaccuracies and biases within the self-reported data. Third, Chronbach's alpha for the perceived social support scale was not high enough for the younger and older group, which may reduce the predictability of perceived social support. Fourth, two different measures of PsyCap were used. Although they are similar in item content, the differences we found in path coefficients may be due to differences in measures rather than differences in age group. Finally, more family and community factors should be considered more carefully in further studies. Despite these limitations, our study explicitly examined the distinct pathway of PsyCap and perceived social support among different age groups of adolescents beyond what has been suggested by previous research and demonstrated how social support and psychological strengths protect the youth from negative outcomes under negative conditions, such as the COVID-19 pandemic.

## Data Availability Statement

The original contributions presented in the study are included in the article/supplementary materials, further inquiries can be directed to the corresponding author/s.

## Ethics Statement

The studies involving human participants were reviewed and approved by the Research Review Committee of the Faculty of Psychology of Southwest University (H20032). Written informed consent to participate in this study was provided by the participants' legal guardian/next of kin. Informed consent form was obtained from all individuals before they completed the study.

## Author Contributions

SC and YZ designed the study and conducted the measurement, performed the statistical analysis, and drafted and revised the manuscript. CD participated in the interpretation of the data and revised the manuscript. PL and WZ participated in the revision of the manuscript. DY conceived of the study, participated in its design, and support the process of collecting data. All authors have read and approved the final manuscript.

## Funding

This study was funded by Fundamental Research Funds for the Central Universities (granting institution: Southwest University, grant number: SWU2009101), Chongqing Planned Social Science Research Program (granting institution: Chongqing Federation of Social Science Circles, grant number 2020TBWT-ZD07), and Graduate Scientific Research and Innovation Foundation of Chongqing (granting institution: Southwest University, grant number CYS20093).

## Conflict of Interest

The authors declare that the research was conducted in the absence of any commercial or financial relationships that could be construed as a potential conflict ofinterest.

## Publisher's Note

All claims expressed in this article are solely those of the authors and do not necessarily represent those of their affiliated organizations, or those of the publisher, the editors and the reviewers. Any product that may be evaluated in this article, or claim that may be made by its manufacturer, is not guaranteed or endorsed by the publisher.

## Footnotes

^1^Given the SE of skewness, we can calculate its corresponding *z*-score. In the case of perceived social support, the skewness *z*-value is −6.9 for the younger group, while the skewness z value is 2.3. If we assume the null hypothesis of skewness *z*-value difference between two groups is zero, the difference in skewness *z*-values is about 4.6 standard units apart, which should be statistically significant at 0.01 level, indicating the skewness is different across these two groups.
